# Element-specific ultrafast lattice dynamics in FePt nanoparticles

**DOI:** 10.1063/4.0000260

**Published:** 2024-11-08

**Authors:** Diego Turenne, Igor Vaskivskyi, Klaus Sokolowski-Tinten, Xijie J. Wang, Alexander H. Reid, Xiaozhe Shen, Ming-Fu Lin, Suji Park, Stephen Weathersby, Michael Kozina, Matthias C. Hoffmann, Jian Wang, Jakub Sebesta, Yukiko K. Takahashi, Oscar Grånäs, Peter M. Oppeneer, Hermann A. Dürr

**Affiliations:** 1 Department of Physics and Astronomy, Uppsala University, Box 516, 75120 Uppsala, Sweden; 2 Department of Complex Matter, Jozef Stefan Institute, Jamova 39, Ljubljana SI-1000, Slovenia; 3 Faculty of Physics and Centre for Nanointegration Duisburg-Essen, University of Duisburg-Essen, Lotharstrasse 1, 47048 Duisburg, Germany; 4 Accelerator Division, SLAC National Accelerator Laboratory, 2575 Sand Hill Road, Menlo Park, California 94025, USA; 5 Faculty of Physics, University of Duisburg-Essen, 47048 Duisburg, Germany; 6 Department of Physics, TU Dortmund University, 44227 Dortmund, Germany; 7 Linac Coherent Light Source, SLAC National Accelerator Laboratory, 2575 Sand Hill Road, Menlo Park, California 94025, USA; 8 Magnetic Materials Unit, National Institute for Materials Science, Tsukuba 305-0047, Japan

## Abstract

Light–matter interaction at the nanoscale in magnetic alloys and heterostructures is a topic of intense research in view of potential applications in high-density magnetic recording. While the element-specific dynamics of electron spins is directly accessible to resonant x-ray pulses with femtosecond time structure, the possible element-specific atomic motion remains largely unexplored. We use ultrafast electron diffraction (UED) to probe the temporal evolution of lattice Bragg peaks of FePt nanoparticles embedded in a carbon matrix following excitation by an optical femtosecond laser pulse. The diffraction interference between Fe and Pt sublattices enables us to demonstrate that the Fe mean square vibration amplitudes are significantly larger that those of Pt as expected from their different atomic mass. Both are found to increase as energy is transferred from the laser-excited electrons to the lattice. Contrary to this intuitive behavior, we observe a laser-induced lattice expansion that is larger for Pt than for Fe atoms during the first picosecond after laser excitation. This effect points to the strain-wave driven lattice expansion with the longitudinal acoustic Pt motion dominating that of Fe.

## I. INTRODUCTION

Future magnetic data storage media will require magnetic nanoparticles with stable ferromagnetic order at diameters of only 10 nm and smaller.^1^ In this respect, granular thin films of the L1_0_-ordered phase of FePt displaying perpendicular magnetic anisotropy (along the out-of-plane c-axis) are one of the most suitable storage media. The FePt nanoparticles composing such granular materials remain ferromagnetic as a result of the strong magnetocrystalline anisotropy needed to overcome the superparamagnetic limit.^2–5^ However, a byproduct of strong magnetocrystalline anisotropy is the large magnetic field required to reverse the nanoparticle magnetization. Applications strive to reduce the magnetic switching field by locally heating the nanoparticles above their Curie temperature with a laser in order to thermally assist the switching, a technique known as heat-assisted magnetic recording.^6^

**FIG. 1.**
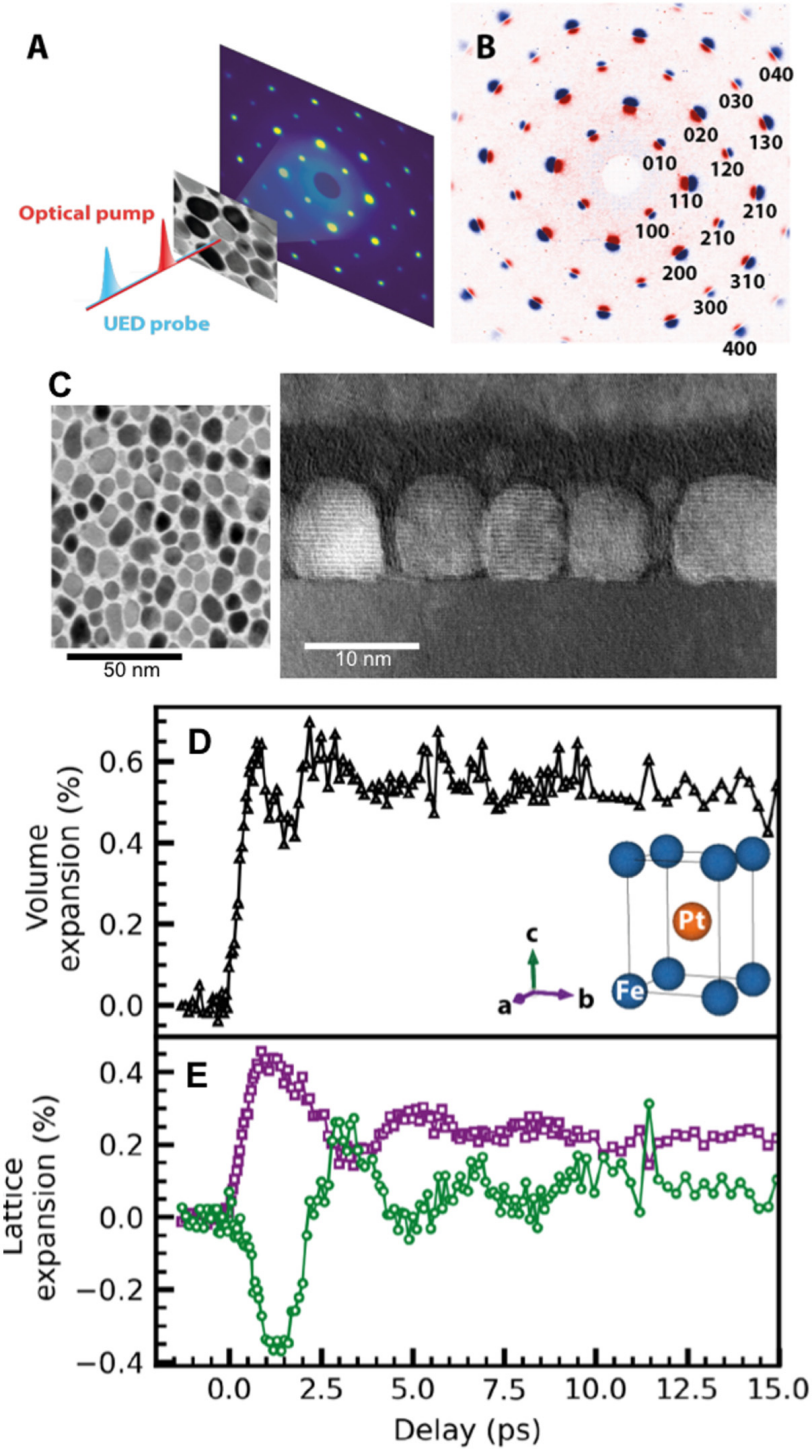
Average lattice expansion. (a) Schematic of the experimental UED pump-probe setup in transmission geometry with optical pump and UED probe beams incident along the FePt c-axis direction [see inset in panel (d)]. (b) Difference between diffraction patterns at 1 ps and at negative delay. The color coding is such that red/blue implies an intensity increase/decrease amounting to about 5%–10% of the peak intensity in (a) depending on the Bragg order (see Fig. 5). (c) High-resolution transmission electron microscopy (TEM) images of the nanoparticles assemblies in the top (left) and side (right) views. (d) Average lattice volume evolution. (e) Temporal evolution of the in-plane (a and b crystallographic directions) lattice spacing in violet and the out-of-plane (c crystallographic direction) atomic spacing in dark green.

The magnetization dynamics of FePt nanoparticles following optical femtosecond (fs) laser excitation has been the subject of various studies resulting in the observation of sub-picosecond demagnetization,^7,8^ element-specific spin dynamics,^9^ and even all-optical magnetic switching.^10^ However, much less is known about the ultrafast lattice response, which has been a topic of attention from theoretical work^11^ and could only recently be experimentally addressed using ultrafast x-ray and electron scattering. Reid *et al.*^12^ showed that the response of suspended 13-nm FePt nanoparticles is characterized by a lattice expansion along the Fe and Pt layers (a, b directions in Fig. 1) accompanied by a contraction of the lattice spacing in the perpendicular direction (c direction in Fig. 1). This reflects a magnetostrictive stress on the lattice due to the laser-induced quenching of the magnetic order.^12^ Key of such studies is that the nanoparticle lattice is free to follow the intrinsic stress buildup within the particles. For instance, FePt nanoparticles with their lattice spacing along the Fe/Pt layers locked into that of a supporting substrate still react to magnetostrictive stress via a lattice contraction along the perpendicular direction.^13^

Here we address the question if the observed changes of the FePt nanoparticle lattice are uniform for the Fe and Pt sublattices. Utilizing the constructive and destructive interference of scattering from both atomic sublattices for Bragg peaks with even and odd sums of Miller indices, respectively, we show that the Pt sublattice expands faster than the Fe sublattice. We correlate this observation with element-specific measurements of the temporal variations observed in mean square vibrations and Brillouin zone boundary phonon occupations.

## II. EXPERIMENT

Single crystalline L1_0_ FePt was grown epitaxially onto a single-crystal MgO(001) substrate by co-sputtering of Fe, Pt, and C.^14^ This resulted in FePt nanoparticles of approximately cylindrical shape with heights of 6 nm and diameters in the 4–12 nm range with an average of 7.1 ± 1.8 nm as corroborated with transmission electron microscopy [Fig. 1(c)]. Due to the single-crystalline substrate, the FePt nanoparticles are identically oriented with the a and b crystallographic directions along the MgO surface and the c-axis perpendicular to it. The volume in-between nanoparticles is filled with glassy carbon at 30% volume fraction. Subsequently, the MgO substrate was chemically removed and the FePt-C films were floated onto copper wire mesh grids with 100 *μ*m wide openings.

The dynamic lattice response of FePt was measured by ultrafast electron diffraction in a transmission geometry [see Fig. 1(a)] with 3.6 MeV electrons from the SLAC ultrafast electron diffraction source.^15^ The pump-probe experiments described below were carried out at room temperature with 1.5 eV/50 fs laser excitation at a nominal pump fluence at a normal incidence of 4 mJ/cm^2^. To keep the deposited energy density constant, the incident laser pulse energy was adjusted to account for the change in beam spot size and sample reflectivity when changing the sample tilt angle.

To meet the Bragg condition for different lattice reflections, the film was rotated around axes normal to the probe beam. Due to geometrical reasons, rotation angles were limited to 45° from normal incidence. Measurements made at normal incidence, with the [001] c-axis parallel to the electron beam, showed changes in the diffraction pattern displayed in Fig. 1(b) as the difference of the pattern at 1 ps pump-probe time delay with respect to that obtained before time zero, i.e., when the optical pump pulses arrive after the electron probe pulses. The Bragg peak positions were determined using a fit of two-dimensional Gaussian profiles to the experiments.^12^ While at normal electron incidence, we probe Bragg peaks along the a,b-crystal axes [see inset of Fig. 1(d)], also c-axis Bragg peaks are accessible when the lattice was tilted away from [001] normal incidence direction. Following Ref. 12, we collected different Bragg reflection position and intensity data after a rotation of the film about the [100] a-axis to a point where the [111] reflections were easily visible. The observed time evolution data of Bragg peaks with different projections along the out-of-plane direction are used to reconstruct the [001] c-axis Bragg intensity following Ref. 12. The results are shown in Fig. 1(e) together with the determination of the average unit cell size variation in Fig. 1(d).

The data displayed in Fig. 1 resemble those obtained for larger FePt nanoparticles.^12^ They show an initial lattice expansion along the a- and b-axes as well as a concomitant c-axis reduction of the lattice spacing due to the reduction of the magnetostrictive stress following a laser-induced ultrafast quenching of the ferromagnetic order.^12^ This leads to a long-lasting average increase in the FePt unit cell during approximately 1 ps after laser excitation followed by a volume-conserving breathing mode at a frequency given by the time it takes an acoustic lattice strain wave to move through the nanoparticles.^12^

## III. RESULTS

### A. Element-specific mean square displacement dynamics

Mean square displacements are a way to measure the vibrations of atoms around their equilibrium positions in a crystal, unveiling the energy stored in the lattice. Within the context of ultrafast lattice dynamics, they serve as an ultrafast proxy for the energy stored in the lattice, making them an ideal tool for tracking the energy flow in an excited, out-of-equilibrium system. The mean square displacements of atoms affect the scattering intensity through the Debye–Waller factor, *M*. In the case of FePt, the integrated intensity of the Bragg peaks is dependent on the Debye–Waller factors of both iron, *M_Fe_*, and platinum, *M_Pt_*. The Debye–Waller factor for diatomic species like FePt has been widely studied in a static regime,^16–20^ but their analysis of Debye–Waller factors in a pump-probe scheme remains constrained for either mono-atomic species^21,22^ or by using lattice temperatures without a clear distinction of the different chemical species.^23–25^

**FIG. 2.**
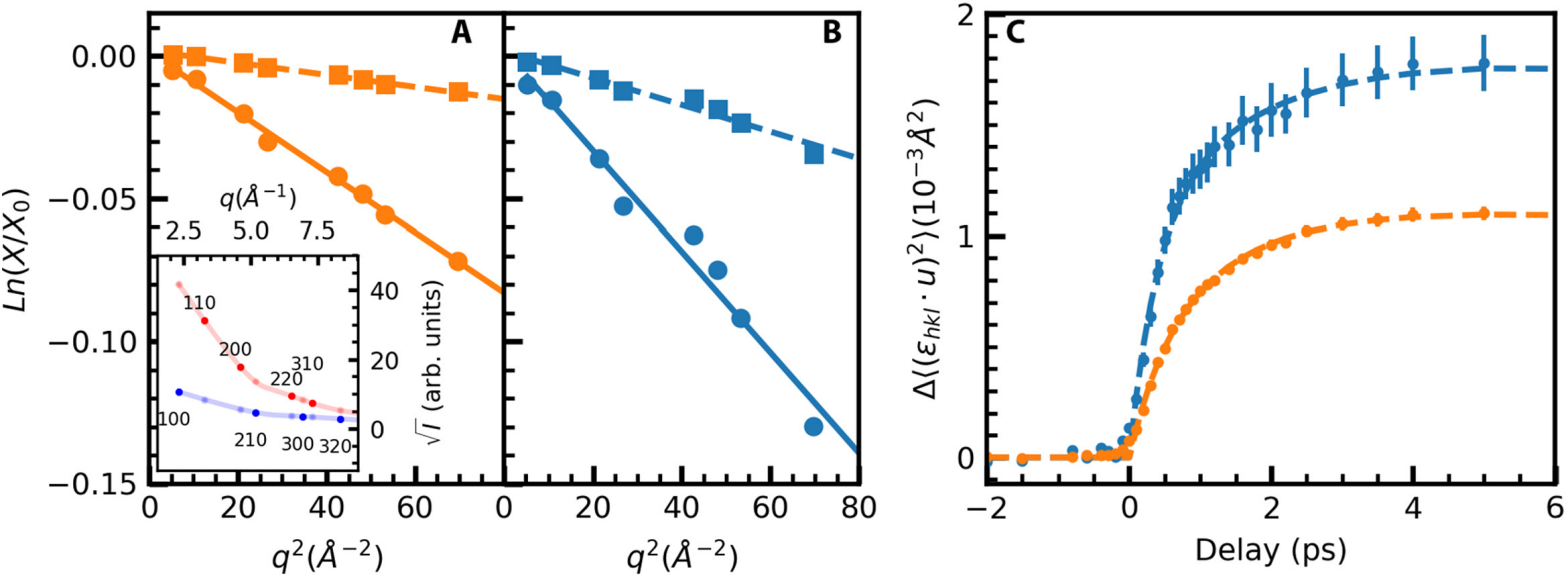
Debye–Waller dynamics. (a) Semi-logarithmic plot of $X=\sqrt{I_{\text{even}}}+\sqrt{I_{\text{odd}}}$ vs *q*^2^ as described in the text. *X*_0_ denotes the values before laser excitation. The slope of the straight-line fits represents the change in Debye–Waller factor, *M_Pt_*, at time delays of 0.2 ps (squares) and 3 ps (circles) relative to before laser excitation. The inset displays the measured intensities of the respective Bragg peaks for *h* + *k* + *l* even (red line and symbols) and odd (purple line and symbols). The light symbols indicate the interpolated intensities as described in the text. (b) Same as in (a) but for $\sqrt{I_{\text{even}}}-\sqrt{I_{\text{odd}}}$ resulting in the Debye–Waller factor, M_Fe_. (c) Temporal evolution of the change in mean square displacements, $\Delta \langle {\left(\varepsilon_{\text{hkl}}\cdot u\right)}^{2}\rangle$, projected onto the direction $\varepsilon_{\text{hkl}}$, of the reciprocal lattice vector $q_{\text{hkl}}$. Shown are data for Pt (orange symbols) and Fe (blue symbols) with the fits described in the text indicated by dashed lines.

Here, we separate the Debye–Waller factors for Fe and Pt atoms using the interference of waves from both atoms with relative phase factors of (−1)^*h+k+l*^ depending on the selected [*hkl*] Bragg diffraction. Constructive and destructive interference between Fe and Pt occurs when *h + k + l* is even and odd, respectively. For a Bragg peak with a reciprocal lattice vector $q_{\text{hkl}}$ and atomic displacements, **u**, the Bragg peak intensity $I_{\text{hkl}}$ is given by^26^

$$I_{\text{hkl}}={\left|{e^{-M_{Pt}}F}_{Pt}\left(q_{\text{hkl}}\right)+{\left(-1\right)}^{h+k+l}\;{e^{-M_{Fe}}F}_{Fe}(q_{\text{hkl}})\right|}^{2},$$ (1)

where $M_{Fe,Pt}=-\frac{1}{2}\left\langle {\left(q_{\text{hkl}}\cdot u_{Fe,\;Pt}\right)}^{2}\right\rangle$ and $\left\langle \ldots \right\rangle$ denotes the Brillouin zone average. The large wavevector transfers in UED experiments enable observation of multiple Bragg peaks in the same experimental geometry. We can, therefore, use the measured Bragg intensities to separate the scattering contributions of Fe and Pt atoms in Eq. (1). The inset of Fig. 2(a) shows the q-dependence of $I_{\text{hkl}}$ measured in equilibrium, i.e., before any laser excitation occurs. It is of course possible to calculate the atomic scattering amplitudes and use them to extract Debye–Waller factors from the data.^26^ Here, however, we use a more intuitive approach leading to identical results. If we interpolate $I_{\text{even}}$ and $I_{\text{odd}}$ to the same reciprocal lattice vectors, ***q***, we can use Eq. (1) to obtain

$$\begin{array}{c} {e^{-M_{Fe}}\;2I_{e}F}_{Fe}\left(q\right)=\sqrt{I_{\text{even}}}-\sqrt{I_{\text{odd}}}\;,\\ {e^{-M_{Pt}}\;2I_{e}F}_{Pt}\left(q\right)=\sqrt{I_{\text{even}}}+\sqrt{I_{\text{odd}}}, \end{array}$$ (2)

where *I_e_* is the electron scattering amplitude.^26^ Figure 2 shows the corresponding evaluation of the Debye–Waller factors M_Fe,Pt_ from Bragg peak intensities in our UED data. We can eliminate the term ${2I_{e}F}_{Pt,Fe}\left(q\right)$ from Eq. (2) by normalizing to the values of $\sqrt{I_{\text{even}}}\pm \sqrt{I_{\text{odd}}}$ in equilibrium, i.e., before laser excitation. For each time delay [delays of 0.2 and 3 ps are shown in Figs. 2(a) and 2(b)], we obtain ${\Delta M}_{Fe,Pt}=-\frac{1}{2}q^{2}\Delta \langle {\left(\varepsilon_{\text{hkl}}\cdot u_{Fe,\;Pt}\right)}^{2}\rangle$, i.e., the change of $M_{Fe,Pt}$ relative to equilibrium, as the slope of a log-plot of $\sqrt{I_{\text{even}}}\pm \sqrt{I_{\text{odd}}}$ normalized to their equilibrium values vs *q*^2^. Here $\varepsilon_{\text{hkl}}$ is a unit vector pointing toward the Bragg peak of order hkl.

Figure 2(c) shows the determined changes of Fe (blue symbols) and Pt (orange symbols) mean square displacements, $\Delta \left\langle {\left(\varepsilon_{\text{hkl}}\cdot u\right)}^{2}\right\rangle$, projected onto the direction, $\varepsilon_{\text{hkl}}$, of the respective reciprocal lattice vectors, $q_{\text{hkl}}=q_{\text{hkl}}\;\varepsilon_{\text{hkl}}$, vs pump-probe time delay. The observed increase in $\Delta \left\langle {\left(\varepsilon_{\text{hkl}}\cdot u\right)}^{2}\right\rangle$ can be described by two exponentials of the form $A_{1}(1-e^{-t/\tau_{1}})+A_{2}(1-e^{-t/\tau_{2}}).$ The determined fit parameters are summarized in Table I.

**TABLE I.** Summary of the fit parameters for the data shown in Fig. 2(c) using a fit function of the form $A_{1}(1-e^{-t/\tau_{1}})+A_{2}(1-e^{-t/\tau_{2}}).$

	A_1_ (10–4 Å^2^)	τ_1_ (ps)	A_2_ (10–4 Å^2^)	τ_2_ (ps)
$\Delta {\left(u_{Fe}\right)}^{2}$	10.8 ± 2.5	0.38 ± 0.08	8.9 ± 2.0	1.8 ± 0.7
$\Delta {\left(u_{Pt}\right)}^{2}$	4.2 ± 3.3	0.5 ± 0.2	7.3 ± 3.0	1.3 ± 0.4

### B. Wavevector resolved phonon dynamics

Insight into the mechanism by which energy is transferred to the lattice to and from electronic and spin degrees of freedom needs wavevector and time-resolved information about the evolution of the phonon populations after laser excitation. The primary source of lattice heating is the hot electron bath that gets excited directly by the laser. The subsequent energy transfer to phonons takes place via electron–phonon scattering events that show a pronounced wavevector dependence, i.e., Brillouin zone boundary phonons are often preferentially populated under the non-equilibrium conditions following ultrafast laser heating.^27^ Diffuse electron diffraction has been used as a unique tool to directly probe the wavevector dependence of such transient phonon populations (e.g.,, Refs. 28–31). Here we extend diffuse scattering to the case of FePt with the aim of separating the non-equilibrium motion of Fe and Pt atoms for selected phonon modes.

**FIG. 3.**
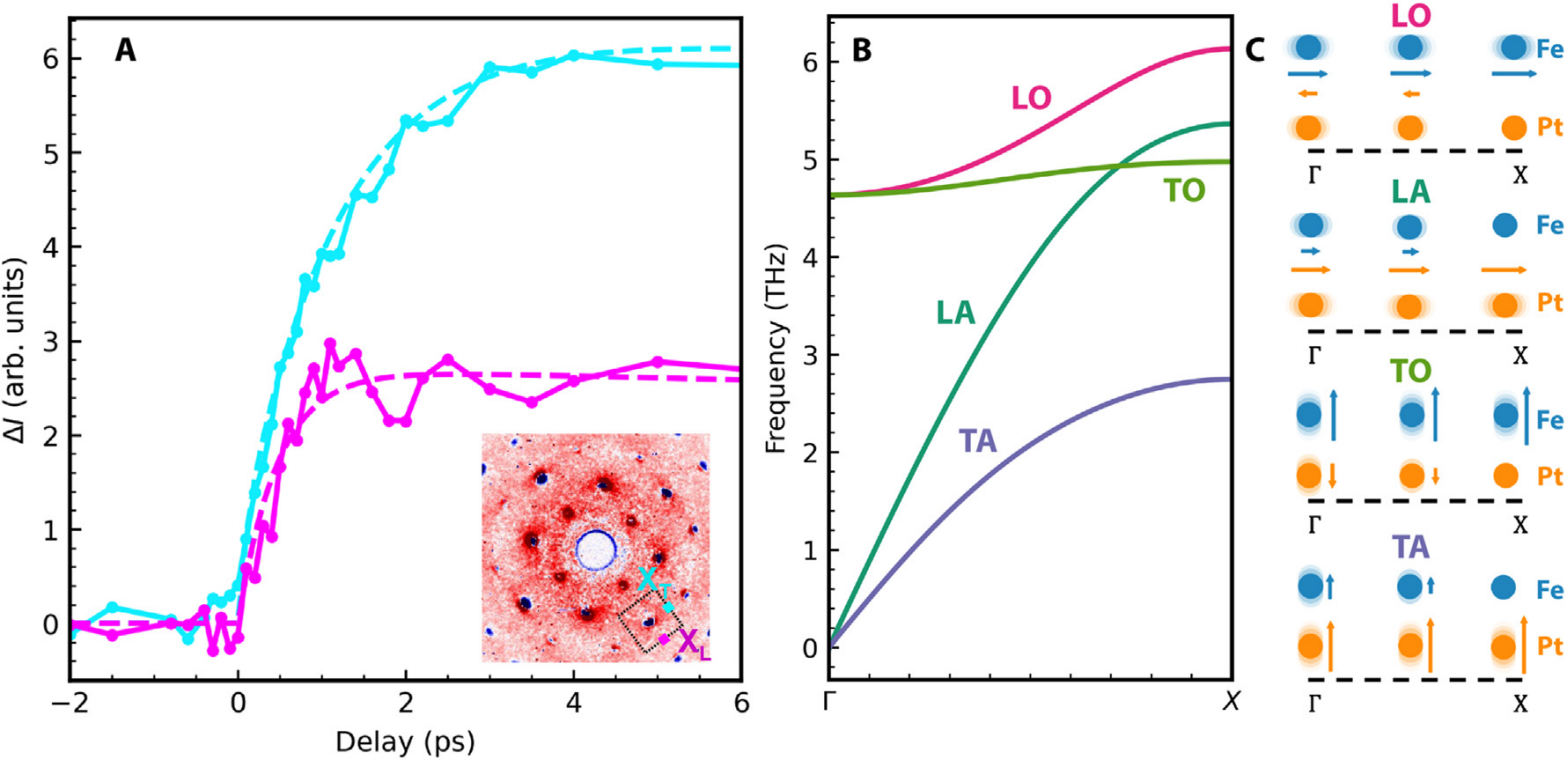
Temporal evolution of diffuse scattering. (a) Diffuse scattering for two X points around the 200 Bragg peak, the regions of interest have been marked in the inset. (b) Phonon dispersions along the ΓX direction for the phonon modes detected in the UED experiment of Fig. 1. (c) Illustration of the calculated phonon eigenvectors of the respective modes at three wavevectors along the ΓX direction. Symmetry dictates that at the Brillouin zone boundary X-point only either Fe or Pt atoms are displaced for each mode.

Figure 3 shows the time-resolved diffuse electron scattering for FePt along the ΓX direction in reciprocal space, which corresponds to the a and b crystallographic axes in the inset of Fig. 1(c). For FePt, we can write the diffuse scattering intensity following Ref. 32 as the sum over the phonon modes for each wavevector k in the FePt Brillouin zone, i.e.,

$$I_{DS}\left(q\right)=\sum_{j}\frac{1}{\omega_{k,j}}\left(n_{k,j}+\frac{1}{2}\right){\left[\frac{F_{Pt}(q)}{\sqrt{m_{Pt}}}{\left(q\cdot e_{k,j}^{Pt}\right)}^{2}\pm \frac{F_{Fe}(q)}{\sqrt{m_{Fe}}}{\left(q\cdot e_{k,j}^{Fe}\right)}^{2}\right]}^{2},$$ (3)

where $m_{Pt,\;Fe}$ are the atomic masses, $F_{Pt,\;Fe}(q)$ are the electron scattering form factors, and $e_{k,j}^{Pt,\;Fe}$ are the phonon eigenvectors for Pt and Fe atoms. $\omega_{k,j}$ and $n_{k,j}$ describe energy and occupation number, respectively, for phonons at wavevector k and branch j. The wavevector k is defined within the Brillouin zone centered on the Bragg peak at a reciprocal lattice vector $q_{\text{hkl}}$. The wavevector transferred in the diffuse scattering process is given by $q=q_{\text{hkl}}+k$. Plus and minus signs correspond to even and odd Bragg orders, respectively.

From a diatomic chain model, it is straightforward to see that at the X-point of the Brillouin zone boundary, phonon modes have eigenvectors where either Fe or Pt atoms are at rest. It follows that the corresponding $e_{k,j}^{Pt,\;Fe}$ shown in Eq. (3) must be zero. This is reproduced in the calculations shown in Figs. 3(b) and 3(c) that were performed following Ref. 27.

The data in Fig. 3(a) can be described by exponential increases of the form $A\left(1-e^{-t/\tau }\right)$. We obtain for the X-points marked in the inset of Fig. 3(a) the fit parameters summarized in Table II, where the term ${\left(\varepsilon \cdot e_{L,\;T}^{Pt,Fe}\right)}^{2}$ describes the square of directional cosines for phonons with longitudinal (L) and transverse (T) polarization at the two X-points. This demonstrates that measurements at the X_L_ and X_T_ points are sensitive mainly to longitudinal and transverse phonon polarizations, respectively.

**TABLE II.** Summary of the fit parameters for the data shown in Fig. 3(a) using a fit function of the type $A\left(1-e^{-t/\tau }\right)$. The terms ${\left(\varepsilon \cdot e_{L,\;T}^{Pt,Fe}\right)}^{2}$ describe the directional cosines for the corresponding phonon polarization at regions X_L,T_ marked in the inset of Fig. 3(a).

	A (10–3 arb. units)	τ (ps)	${\left(\varepsilon \cdot e_{L}^{Pt,Fe}\right)}^{2}$	${\left(\varepsilon \cdot e_{T}^{Pt,Fe}\right)}^{2}$
$X_{L}$	2.7 ± 0.1	0.47 ± 0.06	1.0	0.0
$X_{T}$	6.0 ± 0.1	0.97 ± 0.4	0.07	0.9

### C. Element-specific lattice expansion

Here we describe an extension of the average lattice expansion for FePt nanoparticles beyond what is shown in Fig. 1 and what has been reported so far.^12^ We confine ourselves to normal incidence measuring the a, b lattice expansion depicted in Fig. 1(b). The interference of the scattering amplitudes from Fe and Pt atoms described in Eq. (1) will also allow us to corroborate if the observed lattice expansion is the same for both sublattices.

**FIG. 4.**
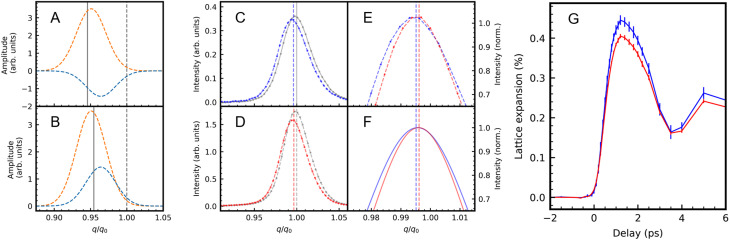
Different lattice expansion of Fe and Pt sublattices. (a) and (b) Cartoon of relative contributions from Fe (light blue) and Pt (orange) sub-lattices to the scattered electron wavefunction at odd (a) and even (b) Bragg orders when the Fe sub-lattice is displaced less than that of Pt. Only when the Fe and Pt lattice spacings are different will the resulting odd and even Bragg peak intensities (marked by the vertical black solid lines) occur at different wavevectors. (c) Measured Bragg peak intensity profile for the 300 order before laser excitation (gray) and at a pump-probe time delay of 1 ps (blue). (d) Measured Bragg peak intensity profile for the 310 order before laser excitation (gray) and at a pump-probe time delay of 1 ps (red). (e) Comparison of the measured 300 (blue) and 310 (red) Bragg peak profiles close to the peak maxima. (f) Comparison of the calculated 300 (blue) and 310 (red) Bragg peak profiles for a Pt sublattice expansion of 0.42% and an Fe expansion of 0.37%. (g) Measured lattice expansion for even (red line and symbols) and odd (blue line and symbols) Bragg orders. An extended dataset is shown in Fig. 5. The error bars contain uncertainties related to the fits (lines) to the data (symbols) shown in panels (c)–(e) and their variation with the Bragg order shown in Fig. 5.

The situation is schematically depicted in Figs. 4(a) and 4(b), where the scattering amplitudes are illustrated for odd and even Bragg orders, respectively. If the lattice expansion is the same for Fe and Pt sublattices, the corresponding odd and even Bragg peaks will be shifted the same amount from the equilibrium lattice position marked by the gray dashed line. If, however, the Pt sub-lattice (orange dashed lines) expands more than the Fe sub-lattice (blue dashed lines), the intensity maxima of the interfering scattering amplitudes for odd and even Bragg orders [indicated by black vertical lines in Figs. 4(a) and 4(b)] will no longer match each other. For destructive interference at odd Bragg orders [Fig. 4(a)], the Bragg intensity maxima will shift to lower values of q/q_0_ than for the constructive interference at even Bragg orders [Fig. 4(b)].

**FIG. 5.**
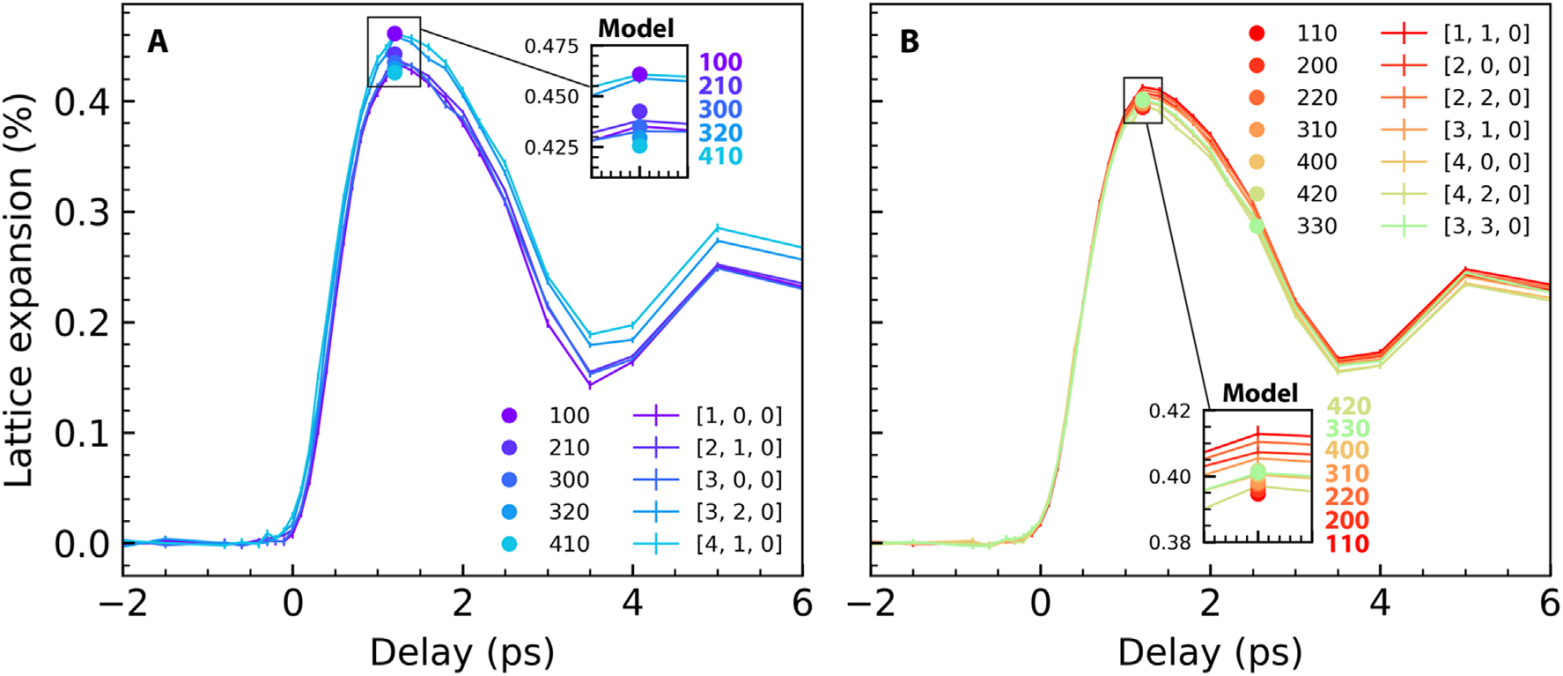
Peak positions as a function of time delay. Measured lattice expansion for (a) odd Bragg orders and (b) even Bragg orders. The average lattice expansions are shown in Fig. 4(g). The calculated intensity maxima of the individual Bragg orders for the model described in the text with a Pt sub-lattice expansion of 0.42% and an Fe sub-lattice expansion of 0.37% are shown as solid circles in the insets.

Although the shift between odd and even orders is relatively small, it can be clearly seen near the Bragg peak intensity maxima in Figs. 4(e) and 4(f). The peak intensity is normalized to remove Debye–Waller attenuation effects seen in Figs. 4(c) and 4(d) and described in Sec. III A. We can model the Bragg peak shift observed in Fig. 4(e) with a Pt sublattice expansion of 0.42% and an Fe expansion of 0.37% as shown in Fig. 4(f). An extended dataset together with the predicted intensity maxima is shown in Fig. 5. This leads to an average peak shift of all measured odd [Fig. 5(a)] and even [Fig. 5(b)] Bragg peaks.

## IV. DISCUSSION

There are several aspects related to FePt nanoparticles that make them unique candidates to study the non-equilibrium interplay between electronic, magnetic, and lattice degrees of freedom. While optical and x-ray pump-probe studies have focused on electron and spin thermalization times following laser heating,^12,33^ our observation of a unit-cell volume expansion in Fig. 1(d) highlights the intricate link behind these processes. The observed timescale for this lattice expansion indicates that it is driven by acoustic strain waves. We have previously observed the longitudinal acoustic (LA) phonons that form the coherent phonon wave packets^34^ driving this expansion in analogy to the THz strain wave propagation in ultrathin Fe films.^35^ The lattice stress driving the expansion can be twofold: (1) electronic stress due to the non-equilibrium population of electronic levels and (2) increased mean square lattice displacements (“heating”) due to electron–phonon energy transfer following femtosecond laser excitation. We showed in Ref. 35 that (1) can displacively launch LA phonon wavepackets. Process (2) proceeds with the characteristic timescale of electron–phonon coupling involving mainly LA modes and to a lesser degree optical phonon modes.^12^ Using the known speed of sound for LA phonons of 4.6 nm/ps^34^ together with the average 7-nm nanoparticle diameter, we find that the lattice expansion rise time of ∼0.8 ps in Fig. 1(d) corresponds to the strain wave traversing about half of a nanoparticle. This is a reasonable estimate since such strain waves originate at the nanoparticle perimeter and propagate inwards leaving an expanded lattice behind.^35^

The unit cell volume increase by more than 0.4% [see Fig. 1(d)] could also significantly alter the electronic structure and affect electron–phonon coupling and energy transfer as observed previously for Ni.^29^ We should, therefore, look for evidence in this direction for the FePt nanoparticle system. An obvious candidate is the fast timescale observed in the longitudinal phonon populations probed in Fig. 3(a) at the *X*_L_-point. We determined the rise time constant to 0.47 ± 0.06 ps (see Table II); however, a saturation-like leveling off is observed at longer times closer to that also evident in the unit cell expansion data [Fig. 1(d)]. It is, therefore, conceivable that the two effects are linked. Inspection of the phonon dispersions close to the *X*-point in Figure 3(b) shows that two longitudinal modes can be detected in our UED geometry, one optical (LO) and one acoustic (LA) mode. They both reach relatively similar frequencies at the *X*-point. However, for the LO mode, only Fe atoms vibrate while the LA mode is characterized by only Pt vibrations [see Fig. 3(c)]. From our measurements alone, we cannot differentiate if one of the two modes is preferentially occupied; however, calculations in Ref. 27 favor a stronger electron–phonon coupling and, thus, mode occupation for the LO mode. Such an assignment with the correspondingly stronger Fe vibration amplitude would also agree with the observed initial increase in the mean square displacements especially for Fe atoms in Fig. 2(c) that occur on a similar timescale.

On longer timescales beyond 1 ps, we observe a slower increase in the mean square displacements in Fig. 2(c) as well as the population of a X-point phonon mode with transverse polarization in Fig. 4(a). Both could be related and are caused by a reduced electron–phonon coupling, possibly in part due to the unit cell expansion. While we cannot rule out from Fig. 3 that the LO mode becomes populated and contributes to the diffuse scattering signal, the larger density of states observed for the TO mode seems to give this mode the preference to contribute at least to the mean square displacement increase in this time range.

The observed unequal expansion of Fe and Pt sub-lattices may be traced back to the strain-induced lattice expansion of FePt nanoparticles. Strain waves propagate with the speed of sound for LA phonons. In FePt, this is about 4.6 nm/ps,^34^ which reproduces the observed oscillation period of the volume-conserving breathing mode in Fig. 1(d) for 7–8 nm diameter particles. However, the first such oscillation cycle will be influenced and is in fact driven by electronic and magnetic stresses.^12,13,35^

Such stresses are related to the non-equilibrium heating of electrons and reduction of the magnetic order that occurs on timescales of just a few 100 fs, i.e., within the transit time of LA strain waves through the nanoparticles. While a detailed modeling of these processes is beyond the scope of the present paper, it is straightforward to imagine that this can lead to an inhomogeneous lattice expansion across a nanoparticle around the 1 ps pump-probe delay time where the maximum a,b-axis lattice expansion is observed [see Figs. 1(d) and 4(g)]. The stronger average lattice expansion for the Pt sublattice (of 0.42%) compared to that of Fe (0.37%) is in line with a larger weight of Pt than Fe to the eigenvectors of this LA mode throughout the Brillouin zone [Fig. 3(c)].

## V. SUMMARY AND CONCLUSIONS

In this work, we have developed a novel approach to study the element specific lattice dynamics of FePt nanoparticles. It is based on using the constructive and destructive interference effects present in multi-atomic lattices. We show that these effects can be utilized based on the simultaneous access to multiple Bragg peaks in ultrafast relativistic electron diffraction. We demonstrated that the method allows us to separate Fe and Pt mean square displacements and corroborated the assignment with diffuse electron diffraction measurements. We identified an inhomogeneous ultrafast lattice expansion that is larger for the Pt than the Fe sublattice, possibly driven by coherent longitudinal acoustic phonon wave packets.

## Data Availability

The data that support the findings of this study are available from the corresponding author.
